# Supplementary material for: Are carbon emissions trading and green financial instruments synergistic? -Comprehensive quantitative research based on content analysis
Source: PLoS One. 2024 Mar 7;19(3):e0298601. doi: 10.1371/journal.pone.0298601 (PMC10919601; doi:10.1371/journal.pone.0298601)
Supplement: S1 Data — (ZIP) [file pone.0298601.s001.zip › Data/地级市Z_F工作报告.zip!/║╙─╧/╨┬╧τ╩╨/└┤╫╘╣1⁄2╓┌║┼í╛┬φ┐╦╩2╛▌═°í┐_2015.docx]

各位代表：

现在，我代表市人民政府，向大会作工作报告，请予审议，并请各位政协委员和列席的同志提出意见。

一、2014年工作回顾

2014年是本届政府的开局之年，面对经济下行和一些领域的突出问题，在省委、省政府和市委正确领导下，在市人大、市政协监督支持下，市政府与全市人民一道，全力以赴稳运行、破难题、谋长远、保民生，较好完成了市十二届人大一次会议确定的主要目标任务，各项事业发展取得新的成绩。

——量的增长有位次。完成地区生产总值1918亿元，增长9.3%，总量和增速均位居全省第6位，增速在全省前移3个位次；规模以上工业增加值870亿元，增长12.3%，增速位居全省第4位，在全省前移8个位次；固定资产投资1842亿元、一般公共预算收入139亿元，总量均位居全省第4位；社会消费品零售总额增长12.8%，增速位居全省第6位。

——调的进程在加快。二三产业比重提高0.2个百分点，粮食总产83.2亿斤，实现十一连增；一般公共预算收入对房地产的依存度降低5个百分点；工业投资对投资增长的贡献率高于全省3个百分点；亿元及以上项目完成投资占全市投资的71.2%，提高0.9个百分点。

——质的提升有成效。城镇化率达到47.58%，高于全省2.4个百分点；城镇居民人均可支配收入、农民人均纯收入分别增长8.5%和10.3%；节能减排完成省政府下达的任务，环境质量逐步改善。

一年来所做的主要工作，可以概括为以下关键字：

一是“稳”，就是全力稳定经济运行。把稳住经济运行作为全年工作的重中之重。明确责任分工，在全省率先出台促进经济持续健康发展的意见，将12个方面、54项稳定经济运行的任务落实到市级领导和责任部门，全力抓好落实。解决突出问题，由市级领导分包300家重点企业，为企业解决土地、用工等问题197个，问题解决率达到96.1%；全年工业用电量增长5.1%，33个工业行业大类中，24个行业增加值实现正增长。强化要素保障，改进新增建设用地指标管理办法，更加注重扶优保强，获批建设用地3.2万亩，获准增加建设用地空间10.3平方公里；加强与金融机构对接，银企洽谈会到位资金182亿元，新增贷款137亿元；深化校企合作办学，300家企业与职业院校建立合作关系。扩大有效投资，开展招商引资百日攻坚活动，成功举办第九届豫商大会，新签约亿元以上项目93个，实际利用外资、到位省外资金总量分别位居全省第4位和第6位；省市重点项目提前1个月完成投资目标，宝钢集团易拉罐等238个亿元以上项目开工，中国电波科技城一期等332个亿元以上项目竣工。

二是“提”，就是推动产业发展提档升级。以产业集聚区为载体，加快培育产业集群，提高产业关联度和整体竞争力。完善规划，结合产业发展现状，出台千百亿产业集群培育三年行动计划，对产业的空间布局、发展重点、努力方向进行优化调整，全市重点发展装备制造、电池及电动车、生物与新医药、食品加工等千亿产业，每个县（市、区）重点培育1—2个超百亿的特色产业集群。龙头带动，借鉴龙头带龙身、龙身找龙头、一条龙引进等招商模式，围绕整合提升电池及电动车产业、北斗系列产品开发应用等，与富士康集团、中兵集团等知名企业对接取得积极成果，新签约产业集群项目41个。获批国家唯一的新能源材料及电池材料战略性新兴产业区域集聚发展试点。重点扶持，集中60%的工业用地和环境容量，重点支持产业集群发展，推动引进项目进驻相对应的产业集聚区。全市装备制造产业产值突破千亿，县域百亿产业集群增加5个，累计达到10个。

三是“破”，就是努力破解发展中的难题。注重通过改革的办法和市场的机制，综合施策，逐步解决发展中的难题。破解房地产业发展难题，在全省率先出台促进房地产市场平稳健康发展的指导意见，成立棚户区（城中村）改造办公室，理顺工作机制，强化推进措施，房地产新开工528万平方米。破解金融难题，坚持化解处置风险与打造诚信环境、拓宽融资渠道并重，优化金融生态环境。设立过桥资金，帮助19家企业完成转贷业务26笔；严厉打击逃废银行债务行为，果断决策、依法处置企业互保、非法集资、投资理财担保公司风险等问题，相关突发事件得到控制；加快企业上市，科隆新能源被上市公司深天地并购，日升数控正在证监会审核，6家企业在新三板挂牌，数量居全省第3位。破解项目开工不足难题，结合行政审批制度改革，强力推进建设项目并联审批，审批时限压缩至34个工作日；加大督导协调力度，提升项目“三率”水平，新晋高速、中心医院东区医院等46个项目的遗留问题正在逐步解决，实施进度加快。破解改革中的难题，第一批重点改革事项顺利推进，233项工作完成阶段性目标。市本级再次取消下放调整审批项目75项，进驻市行政服务中心的项目占保留项目的94%；政府机构改革稳步推进，完成工商、质监和食药监管理体制调整；以破解拆迁、资金难题为重点，抓好新型农村社区建设，全国农村改革试验区通过国家中期评估；推进工商注册制度便利化，各类市场主体增长24%；强化科技创新，争取省重大科技专项4项，国家高新技术企业总数达到69家，均位居全省前列。

四是“谋”，就是谋划事关长远发展的大事。站位全局，梳理个性特征和比较优势，努力抢占新一轮发展的制高点。谋划推进大健康产业发展，发挥旅游、文化、医药等综合优势，谋划推进大旅游、大健康、大文化产业融合发展，与西部控股签订大健康产业项目框架协议；旅游资源整合成效明显，接待国内游客人数、国内旅游收入分别增长13.5%和16.4%。谋划推进新业态发展，利用驻新高校及大学生资源、较为完备的职教体系等比较优势，谋划电子商务等新业态发展，相继实施了区域电子商务基地、基于北斗导航的智能化车辆服务平台研发与示范、小货郎等项目，被列为国家信息消费试点市。加快推进郑新融合，制定专项工作方案，多次与郑州就规划编制、基础设施建设等进行对接，取得积极成效，有力促成郑州与毗邻城市形成组合型大都市区上升为全省战略，省里初步同意郑济快速铁路接入新乡东站。谋划提升城市聚合度，研究推进平原路东延、牧野路北延、市区到南太行的快速通道和城市轨道交通项目，推进市商务中心区规划调整，抓好铁西区域改造，逐步推动城市功能区的高度聚合。中心城区完成拆迁297万平方米，22条城市道路改造建设稳步推进，占道经营等城市管理顽疾正在逐步进行治理。借助“两区”建设，以完善基础设施为重点，加快县城发展，“两区”规划总面积17.9平方公里，完成投资60亿元。

五是“保”，就是进一步保障和改善民生。财政民生支出209.5亿元，占一般公共预算支出的75%，省市承诺为群众办理的实事全面完成。第一期学前教育三年行动计划顺利完成，新建、改扩建幼儿园283所；接收进城务工人员随迁子女就学9968人，实现应入尽入、同城同待遇；资助各类家庭困难学生9万余人次，基本保障了困难家庭孩子不因贫困失学；在全省率先开展校长教师轮岗交流，多元合作办学全面铺开，237所学校结成115个合作体；普通高考本科上线率51%，高于全省8个百分点；职业教育工作受到全国表彰。城镇新增就业9.27万人，农村劳动力转移就业122.6万人次。新农合和城镇居民医保财政补助每人每年均提高40元，新农合参合率达到99.73%，新农合大病保险经验在全省推广。城乡居民基本养老保险、城镇居民基本医疗保险参保人数分别增加2.3万和0.4万人，企业退休人员基本养老金人均月增198元。为城乡低保和农村五保对象发放救助金3.8亿元。建成保障性住房16941套。在省第十二届运动会上，我市金牌数量位居全省第4位，比上届提升5个位次。“单独两孩”生育政策顺利实施，人口自然增长率控制在6.5‰以内。封丘县李庄镇被列为全省黄河滩区居民迁建试点，搬迁户数、人数分别占全省的43.9%和45.7%；全市扶贫搬迁423户、2000人。解决29.3万农村居民和师生安全饮水问题。加强环境保护，拆除改造燃煤小锅炉154台，淘汰黄标车1.3万辆，建成5座污水处理厂；林业生态建设持续推进，成为全省首批生态文明先行示范市建设地区。南水北调中线工程新乡段总干渠正式通水。严厉打击各种制假售假行为，查办案件271起。全国质量强市示范城市创建通过预验收。安全生产实现事故总量、死亡人数和较大以上事故起数“三个下降”，平安建设稳步推进，社会大局和谐稳定。

一年来，我们自觉接受人大监督、政协民主监督和社会各界监督，人大代表建议和政协提案办结率100%。我们深入开展党的群众路线教育实践活动，严格执行“八项规定”和“约法三章”，一批“四风”问题逐步得到解决，“三公”经费支出下降27.7%。我们注重加强自身建设，出台相关办法，规范政府投资项目、政府融资、债务管理、国有资产管理及重大行政决策程序等，提升法治政府建设水平，反腐倡廉取得新进展。国防动员、民族宗教、统计审计、广电新闻出版、外事侨务、人民防空、史志档案、气象地震等工作都取得新成绩。

各位代表！

过去的一年，全市上下戮力同心、克难攻坚，全力应对经济下行，逐步破解了发展中的一些难题，取得了来之不易的发展成果。虽然经济增长与预期目标有一定差距，但仍高于全国、全省1.9和0.4个百分点，继续运行在合理区间，好的发展态势得到持续。这是省委、省政府和市委正确领导的结果，是市人大、市政协监督支持的结果，是全市人民共同努力的结果。在此，我代表市人民政府，向辛勤工作在各行各业的全市人民，向给予政府工作大力支持的人大代表、政协委员、离退休老同志，向驻新官兵和中央、省驻新单位，向所有关心、支持、参与新乡发展建设的社会各界人士和广大海内外朋友表示衷心的感谢！

各位代表！

我们也清醒地认识到，全市发展还存在不少问题，政府工作与群众的期望还有差距。一是经济下行压力较大。部分行业产能过剩，生产经营较为困难；财政收入结构不合理，增速放缓；发展不足的基本市情没有改变，全面建成小康社会的任务十分艰巨。二是产业结构不合理。产业集群化水平不高，龙头带动作用不强，“小”和“散”的问题突出；服务业占比低，农业持续增产、农民稳定增收难度加大，转型升级的步伐需要加快。三是民生领域欠账较多。财政刚性支出增加，收支矛盾突出；群众对教育、医疗、环保、城市管理等领域还有不少不满意的地方；处理非法集资还有大量工作要做，一些领域的潜在风险逐步显现，改善民生和加强社会治理的任务十分繁重。四是自身建设有待加强。政府职能转变、简政放权还不到位，公务员的思想观念、工作作风、能力素质还需要加快适应新常态。少数单位和工作人员的全局意识、责任意识、担当意识、法律意识有待强化，存在为官不为、不敢担当的情况，腐败现象仍时有发生。这些问题首先要从政府自身找原因，我们一定加倍努力，尽快加以解决。

二、2015年工作总体要求和主要目标

今年是全面深化改革的关键年，是全面推进依法治国的开局年，也是全面完成“十二五”规划的收官年，做好全年工作意义重大。

今年政府工作总体要求是：以邓小平理论、“三个代表”重要思想、科学发展观为指导，全面贯彻党的十八大和十八届三中、四中全会精神，贯彻落实习近平总书记系列重要讲话精神，准确把握省委、省政府“三大国家战略规划、一个载体、四个体系、六大基础”的战略重点，按照“一个总目标、两条主线、一个总抓手、四个支撑”要求，以提高经济发展质量和效益为中心，以改革开放和创新驱动为动力，坚持稳中求进，稳中提升，稳中突破，着力扩需求、调结构、创优势、增活力、惠民生、控风险，促进经济平稳健康发展和社会和谐稳定。

经济社会发展主要目标是：地区生产总值增长8.5%，一般公共预算收入增长8.5%，规模以上工业增加值增长11%，全社会固定资产投资增长16%，社会消费品零售总额增长13%，城乡居民收入与经济同步增长，完成省定节能减排目标任务。在确保发展质量和效益的基础上，各项指标要争取更好的结果。

做好全年各项工作，要牢牢把握发展第一要务，深刻认识新常态、主动适应新常态、积极引领新常态：一要融入大局。树立战略思维，自觉在全省乃至全国一盘棋中找定位，把工作切入点和发力点融入新常态下发展的大趋势、上级的大战略和区域经济的大格局，编制好“十三五”规划，掌握发展的主动权。二要突出特色。把握新常态的共性特征，找准自身的个性特点，充分发挥比较优势，抓住布一子而全局活的重点工作不放，努力在专业化分工中做大做强自己。三要遵循规律。决不追求短期行为，不杀鸡取卵，不胡干蛮干，要遵循经济规律，推动科学发展；要遵循自然规律，推动可持续发展；要遵循社会规律，推动包容性发展。四要顺势而为。顺应和跟进大势，因其时，顺其势，行其道，用足机遇、用好优势、用活政策，努力借势取径，借梯登高，变“势”为“事”。

各位代表！

改革发展面临的任务很重，归根结底是责任之重、担当之重、落实之重。只要我们脚踏实地，埋头苦干，在正确的路子上踏石留印，在行之有效的举措上抓铁有痕，就一定能够在新常态下爬坡过坎、行稳致远，开创全市各项事业发展的新局面！

三、2015年重点工作

今年要重点抓好以下方面的工作：

（一）在经济运行上下功夫，推动经济发展提质增效。坚持长短结合，稳定经济运行，防控金融风险，搞好企业服务，不断提升发展的质量和效益。一要全力稳定经济运行。加强要素协调供给，帮助优势企业扩大生产，帮扶暂时困难的企业走出困境，推动过剩产业改造升级和兼并重组；对企业反映集中的资金、用工等问题，要一对一搞好服务。全面落实促进房地产业平稳健康发展的各项措施，努力破解棚户区（城中村）改造难题，开工建设公村城中村改造等20个项目，建设安置房1.8万套；优化房地产结构，促进房地产业与文化、旅游、养老等产业融合发展。二要全力优化金融生态环境。严厉打击逃废银行债务行为，抓好农信社等不良贷款清收，打造诚信环境，增强银企互信；全面加强对担保理财投资类公司经营监管，依法依规、全力做好全顺、腾飞等非法集资案件处置工作，最大限度挽回群众损失；建立完善银企对接长效机制，帮助企业拓宽融资渠道，加大直接融资比重，新三板挂牌企业达到10家以上。三要全面提升企业发展的质量和效益。大力实施创新驱动发展战略，坚持企业的主体地位，发挥驻新高校和科研院所作用，启动科技创新公共服务平台建设行动计划，完善校地对接机制，大力引进高层次人才和紧缺急需人才，推动产学研深度融合，实施重大科技专项10项、科技成果转化项目20项，专利申请量突破3700件。加强企业家交流培训，帮助企业提升发展战略，提高管理水平和应对能力。把质量强市建设作为推动产业升级的重要抓手，严厉打击质量违法行为，深入实施名牌战略，争创省名牌产品10个，争取通过“全国质量强市示范城市”验收。

（二）在产业集群上下功夫，培育新的经济增长点。坚持存量调整与增量提质并重，加快实施电池及电动车、生物与新医药等千亿产业培育行动计划，实施重点突破，加快转型升级。一要争取电池及电动车产业实现新突破。抓住被列入国家新能源材料及电池材料战略性新兴产业区域集聚发展试点的机遇，力争通过富士康项目的引进与实施，整体盘活电池及电动车产业，打造国家级产业基地。抓好新能源汽车推广应用，确保通过国家考核。二要争取生物与新医药产业实现新突破。抓住药号转移和创新药审批的机遇，加快华兰生物、双鹭药业两个百亿园区发展和单克隆抗体、重组人血白蛋白等新产品开发，推动生物与新医药产业加快发展。三要争取北斗产业实现新突破。抓住信息经济特别是北斗产业发展机遇，外引杭州中导公司投资建设北斗产业园，内联22所、新飞电子等企业，加快培育北斗产业集群。四要争取服务业新业态实现新突破。发挥“两区”的载体和引领作用，滚动实施80个服务业重大项目，依托互联网、现代信息技术发展服务型制造、微电商等新业态，推动服务业扩量提质，服务业增加值增长8%以上。五要争取大健康产业实现新突破。抓住国民消费升级的机遇，依托南太行旅游、全市医疗等资源优势和封丘县“中国长寿之乡”等品牌优势，加强与西部控股等企业的对接合作，努力打造河南省养老健康产业发展示范基地。加快基础设施建设，提升管理水平，尽快把南太行建成国家一流的休闲度假区。全市接待游客人数增长10%，旅游总收入增长13%。同时，要抓好新乡市云计算中心建设和运营，提升信息化基础设施建设水平；以智能制造为切入点，培育数字化车间、智能工厂，促进“两化”深度融合。

（三）在整合提升上下功夫，提高区域经济聚合度。把城镇作为区域经济整合提升的重要聚合点，强化基础提升、交通先行、“两区”带动、产城融合，提升整体竞争力。一要谋划推进重要经济“带”。按照“借势航空港、共建大都市、承担大功能、形成大合力”要求，加快郑新融合发展。全力争取我市与“米”字形快速铁路相关项目纳入国家盘子，推进郑州地铁与新乡城市轨道交通系统连接线前期工作，抓好平原示范区、金水（获嘉）产业新城建设。编制以新乡、郑州黄河两岸为主的生态经济产业带规划，加强与沿黄城市对接，争取将沿黄经济产业带建设纳入全省战略。逐步培育郑新、沿黄“十”字经济带，在全省组合型大都市区建设中分担更大功能，发挥更大作用。二要整合提升关键支撑“点”。强化规划在城乡建设中的龙头作用，加快河湖渠水系连通规划等各类规划编制和完善，为建设靓丽城市打下基础。要突出抓好中心城市和县城发展建设，中心城区方面，推进道路互联互通，新建和改造28条城市道路，加快推进平原路东延和牧野路北延，谋划国道107新乡段东移工程和城市轨道交通项目，为大东区整体建设、铁西区域开发、凤泉区与中心城区融合创造条件，提高城市聚合度；把市商务中心区作为拉大城市框架、完善城市功能的重要板块，完成各类规划编制，加大招商力度，培育发展金融服务、总部经济等新业态。推动城市管理重心下移，大力开展市容环境重点整治，突出抓好占道经营、建筑垃圾、违法建设等专项整治行动，加强和规范物业管理，全力推动城市管理工作步入长效化轨道。县城方面，要借助“两区”建设，加快旧商业街区和城中村改造，完成拆迁235万平方米，完成“两区”建设投资60亿元，提升县城综合承载能力。强化“一基本两牵动三保障”，将县城打造成为农业人口转移的主要载体。三要扩大产业辐射带动“面”。突出承接转移、集群发展、完善配套、创新体制、产城互动，推动产业集聚区提档升级，力争超百亿元产业集聚区达到8家以上，就近转移就业50万人以上。加快平原示范区副中心城市、高新区、经开区发展，打造我市区域经济和长远布局的重要支撑。支持靠近中心城区的专业园区调整发展方向，推动达到标准的专业园区晋升产业集聚区。抓住土地利用总体规划调整完善的重要机遇，优化各类用地布局，积极争取上级用地指标，拓展发展空间。

（四）在三农工作上下功夫，加快转变农业发展方式。强化“一稳两转”，通过新型城镇化和农业现代化“两手”发力，推动农业发展走出新路子。一要促进稳粮转型增效。深入实施高标准粮田建设、现代农业产业化集群培育和都市生态农业发展“三大”工程。做好基本农田划定工作，持续推进以小农水重点县、灌区节水改造、中小河流治理为重点的农田水利建设；新增高标准粮田40万亩，粮食产量稳定在80亿斤以上；新增年销售收入10亿元以上的产业化集群3个；稳步推进都市农业生态圈建设，培育农业新业态。大力发展现代畜牧业，新创建标准化养殖场50个。加强农产品质量监管，安全抽检合格率达96%以上。二要构建新型农业经营体系。鼓励发展农民专业合作社、专业大户、农业企业等多种经营主体，逐步构建现代农业经营体系，培育3家以上省级家庭农场；支持供销、邮政系统发展农资连锁经营，健全农业生产社会化服务机制。三要强化科技支撑。抓好国家农业科技园区建设，加快农业科技联盟创建，实施10个以上农业科技成果转化专项；利用农业科技资源优势，促进一、二、三产业融合，推动农业“接二连三”，提升农业附加值。四要加快美丽乡村建设。坚持五规合一，因地制宜、量力而行，分类推进新农村综合发展，重点抓好一类社区建设。全面开展农村人居环境达标村、示范村创建工作，用两年时间基本建立村庄保洁长效机制。加快黄河滩区居民迁建，全市实现5万农村贫困人口稳定脱贫。加快引黄调蓄和抗旱水源工程建设，解决20万农村居民和师生饮水安全问题。新改建农村道路350公里。

（五）在项目建设上下功夫，增强发展后劲。深入实施招商引资和项目带动战略，落实项目识别、审批、推进及督导等工作机制，提高引进项目的科学性、精准性、安全性和转化率。一要提升招商引资工作水平。更加注重招大引强、招新引高、招才引智。围绕主导产业，抓好以大健康为龙头的旅游医疗服务、天光科技为龙头的电子信息、百威啤酒为龙头的食品加工等产业集群招商，力争省级产业集聚区各引进1个10亿元以上的产业集群项目。更加注重与各类商会、协会和沿海开发区、产业园区合作，发挥企业的招商主体作用。全年签约亿元以上项目80个以上，开放型经济指标位居全省前列。完善拓展新乡海关、出入境检验检疫局功能，构建开放平台体系。二要强力推进重点项目建设。在产业集群培育、健康养老、基础设施等领域谋划实施10大工程项目包，全力攻坚，发挥示范带动作用。实施亿元及以上项目600个以上，完成投资900亿元。完成南水北调配套工程主体工程，确保6月底前市区居民吃上优质丹江水。要借助“十三五”规划编制，谋划好事关全局的重大项目和生产力布局，争取一批项目列入国家和省规划。

（六）在深化改革上下功夫，释放发展活力。积极跟进落实中央、省出台的改革举措，确保第一批269项重点改革任务落实。一要深化行政体制改革。继续取消和下放审批事项，深入推进“两集中两到位”工作，强力推进建设项目并联审批，深入开展政务服务标准化试点，努力打造最好的投资环境。二要深化投融资体制改革。整合现有涉企发展资金，研究设立我市产业发展引导基金，用有限的财政资金撬动社会资本；积极推进城市基础设施和社会事业PPP模式示范项目，吸引民间资本参与建设运营。突出以管资本为主加强国有资产监管。推动政府投融资公司转型发展。三要深化财税体制改革。抓好预算管理制度改革，建立市级财政专项资金管理目录，除涉密信息外，所有使用财政资金的单位全部公开预决算。健全地方政府性债务扎口管理等机制。抓好“营改增”试点政策落实，逐步将建筑业、金融业、生活性服务业等纳入试点推行。四要深化农业农村改革。加快农村土地承包经营权、房地一体的农村宅基地和集体建设用地使用权确权登记，让农民吃上定心丸；引导和规范土地承包经营权有序流转，发展多种形式的规模经营，避免“非粮化”，坚决禁止“非农化”；建立农村产权交易市场，拓展交易品种，以农户承包土地经营权、集体林地经营权为主，逐步把农村沉睡的资产变成流动的资本。加快全国农村改革试验区各项工作，努力解决改革成果碎片化问题。五要统筹推进其他重点领域的改革。按照上级统一部署，抓好不动产统一登记、公务用车、医药卫生体制、户籍制度、价格等领域的改革。

（七）在改善民生上下功夫，大力发展社会事业。经济越是下行，财力越是紧张，越要把保障和改善民生放在突出位置。一要千方百计扩大就业。支持以创业带动就业，统筹做好高校毕业生、城镇就业困难人员、零就业家庭等群体就业帮扶，妥善解决化解产能过剩中下岗分流职工再就业问题，城镇新增就业6.85万人；加大治理欠薪和打击非法中介力度，保障劳动者合法权益；抓好农村劳动力培训，新增转移就业3万人。二要完善社会保障体系。推进机关事业单位养老保险制度改革，推动城乡居民与城镇职工养老保险转移接续，完善医疗、工伤、生育保险市级统筹。健全社会救助体系，探索建立留守儿童、留守老人关爱服务体系，努力让每个身处困境者都得到关爱。抓好已建成保障性住房分配入住，新开工各类保障性安居工程22934套。三要努力办好人民满意的教育。大力实施农村学校全面“改薄”和城镇教育资源扩充五年规划，优化学校布局，用三年时间，在主城区新建4所初中、12所小学；到2016年，全市新建、改扩建幼儿园301所，学前三年毛入学率达到88%，基本实现学前教育普及普惠；大力推进农村学校标准化建设，努力解决农村中小学师资力量匮乏问题，做好进城务工人员随迁子女入学和贫困学生资助工作，让所有孩子都有公平的发展机会；提高普通高中教育质量，加快职业教育发展，支持驻新高校壮大。四要提升群众健康水平。加强基层医疗人才培养和医疗机构标准化建设，扩建改造2个县级医院、5个乡镇卫生院、200个村卫生室；新农合财政补助标准人均提高60元。抓好中心医院东区医院、市健康管理与康复中心、河南省干细胞与再生医学产业基地等项目建设。积极推进结防所、妇幼保健院整体搬迁。坚持计划生育基本国策，促进人口长期均衡发展。广泛开展全民健身运动，提高竞技体育水平，增强人民体质。五要加强环境保护。抓好蓝天工程、碧水工程和乡村清洁工程实施，完成“十二五”落后产能淘汰任务，继续加大燃煤锅炉拆除改造力度，开工建设10个污水处理厂，推进6个公园改造提升，抓好重要河道及中小河流综合治理，完成林业生态建设11万亩，完成水土流失治理面积30平方公里，加强地下水资源管理和保护。严厉打击各类环境违法违规行为，努力建设天蓝地绿水净的美好家园，让美丽与发展同行、生态与文明共进。六要统筹推进各项事业发展。抓好全国文明城市创建，弘扬新乡先进群体精神，发出好声音，展示新形象。健全公共交通服务体系，加强出租车行业专项治理，净化客运市场，提高服务质量。加快免费开放基层公共文化体育设施进程，提升公共文化服务水平。加强国防动员和后备力量建设，深入推进富有新乡特色的军民融合发展路子，争创全国双拥模范城“六连冠”。促进民族团结进步和宗教关系和谐，推动防震减灾、统计、史志、外事、侨务、档案、测绘、人防等各项事业全面进步。七要创新社会治理。着眼“双安”、推进“双治”、强化“双基”，加快传统社会管理向现代社会治理转变。推动社会矛盾调处由事后处置向事前预防转变，落实阳光信访、依法逐级走访等信访制度改革措施，用法治思维和方式化解社会矛盾，引导社会组织有序参与社会治理，支持工会、共青团、妇联、工商联等人民团体发挥桥梁纽带作用。妥善化解困难行业、金融及社会领域积累的风险，坚决守住底线。支持应急产业发展，强化应急管理。全面落实安全生产责任体系，加大隐患排查治理力度，强化食品药品质量安全监管，严厉打击各类违法违规行为，确保社会和谐稳定。

各位代表！

面对新常态和全面建成小康社会、全面深化改革、全面推进依法治国、全面从严治党新要求，我们将把加强自身建设、打造良好环境作为保障发展、改善民生的重要任务来抓。

我们一定依法履职。严格执行人大及其常委会决议决定，自觉接受人大法律和工作监督，主动接受人民政协民主监督和社会各界监督。逐步向社会公开人大代表建议和政协委员提案办理结果，更好回应社会关切。“有权不可任性”，要加快编制完成权力清单、责任清单，让政府法无授权不可为、法定职责必须为，让社会法人法无禁止皆可为。建立完善重大决策终身责任追究及责任倒查机制，把公众参与、专家论证、风险评估、合法性审查、集体讨论决定等法定程序落到实处；完成“六五”普法任务，规范行政自由裁量权，加强行政复议和行政应诉，公正文明廉洁执法，提升行政法治化水平，打造法治政府。

我们一定敢于担当。各级领导干部要带头坚持原则，敢于担当、敢于较真、敢抓敢管。各级各部门要站位全局、上下对接，不上交矛盾、不推诿扯皮、不互设工作前提，以敬畏之心、带着责任对待工作和群众。要进一步简化办事程序，提高办事效率，严厉查处工作懒懒散散、松松垮垮、不在状态的单位和个人。

我们一定狠抓落实。巩固党的群众路线教育实践活动成效，把抓落实作为政府工作的生命线，重点工作、重点项目、重要民生工程要定期向人民群众亮进度、晒问题，创造条件让人民监督批评政府，切实兑现向群众的承诺；要强化行政问责，严肃查处各类“中梗阻”问题，确保政令畅通、服务高效、工作落实，不断提高政府的推进力、执行力和公信力。

我们一定勤政廉政。广大公务员特别是领导干部既要干净，又要干事，对为官不为、懒政怠政的单位和个人要公开曝光、严肃问责。要全面落实党风廉政建设主体责任，强化“一岗双责”，规范权力运行，完善“四位一体”服务监督体系，加大审计和审计结果公告力度，推进关口前移。严格执行“八项规定”和“约法三章”，厉行勤俭节约，公用经费定额再压缩5%。要认真践行“三严三实”要求，做到心中有党、心中有法、心中有民、心中有责、心中有戒，做守纪律的干部、讲规矩的干部、敢担当的干部、干干净净的干部。对任何腐败分子、任何破坏发展环境和损害群众利益的行为，我们决不心慈手软，坚决一查到底，坚决清除胡作非为的害群之马！

各位代表！

人民的期待和重托，是我们做好工作的不竭动力，是我们肩负的重大责任和使命。让我们紧密团结在以习近平同志为总书记的党中央周围，在省委、省政府和市委的领导下，团结拼搏，锐意进取，努力开创新乡各项事业发展的新局面，为实现中原崛起河南振兴富民强省做出新的更大的贡献！
